# Atypical Parkinsonism Revealing a Late Onset, Rigid and Akinetic Form of Huntington's Disease

**DOI:** 10.1155/2011/696953

**Published:** 2011-09-07

**Authors:** A. Ciammola, J. Sassone, B. Poletti, N. Mencacci, R. Benti, V. Silani

**Affiliations:** ^1^Department of Neurology and Laboratory of Neuroscience, IRCCS Istituto Auxologico Italiano, Piazzale Brescia 20, 20149 Milan, Italy; ^2^Nuclear Medicine, IRCCS-Ospedale Maggiore, 20122 Milan, Italy; ^3^“Dino Ferrari” Center, University of Milan, 20149 Milan, Italy

## Abstract

Huntington's disease (HD) is a rare hereditary neurodegenerative disorder characterized in over 90 percent of cases by chorea as the presenting motor symptom. We report a 54-year-old male who presented with Parkinsonism as the initial symptom of the disease. Genetic analysis revealed expansion of 40 CAG repeats, and brain MRI showed both severe caudate nuclei and cortical atrophy. Single-photon emission computed tomography (SPECT) imaging of the dopamine transporter showed nigrostriatal pathway degeneration. Here, we also describe his 2 years of clinical followup after ensuing dopaminergic stimulation.

## 1. Introduction

The classical presentation of Huntington's disease (HD) includes midlife onset of dementia, personality disorders, and chorea, with dystonia and Parkinsonism usually appearing later over the course of the disease. Conversely, a minor percentage of patients develop, at the time of disease onset, a rigid and akinetic syndrome described first by Westphal in 1883 [1]. The Westphal's variant frequently occurs in juvenile onset HD cases characterized by large CAG expansions, whereas it very rarely can be observed among patients who display first symptoms after age 50 (late-onset HD), usually having low CAG expansions [2]. These rare cases are worth describing, because such nonclassical clinical features may present a diagnostic problem, sometimes hindering the clinical diagnosis for HD.

In the present study, we report clinical and functional neuroimaging data of an HD patient with a late-onset, rigid, and akinetic presentation.

## 2. Case Presentation

A 70 year-old man was admitted to our department in November 2005 with rising anxiety and severe gait difficulty. He had experienced depression first at 50 years of age, and beginning at 54 years of age, he developed a progressive walking difficulty characterized by bradykinesia, gait freezing, and loss of balance. 

He had no history of encephalitis or toxin exposure, and he had never been treated with neuroleptics or other antidopaminergic drugs. Familiar anamnesis evaluation disclosed that his brother, who died at 66 years of age, suffered from a choreic disorder, whereas his father, who had developed walking difficulties, died at the age of 80. The family history was otherwise negative. 

The patient was evaluated with the unified Huntington disease rating scale (UHDRS part I score = 50) and the unified Parkinson disease rating scale (UPDRS motor score = 27) by a neurologist with expertise in HD (A. Ciammola). He had moderate hypomimia, slurred and slow speech, and symmetric rigidity with “cog-wheeling” at the four limbs. We also observed dystonic posturing of his right hand. No rest or postural tremor was present. Choreic movements of the face and tongue were rarely observed, and his UHDRS chorea subscore was 2. The patient tended to adopt a slightly stooped posture, and his postural reflexes were severely impaired. His walking showed festination, propulsion, and bilateral reduction in arm swing. Cognitive functions were moderately impaired (UHDRS part II = 107.8). Beck depression inventory (BDI) score was 26. 

**Figure 1:**
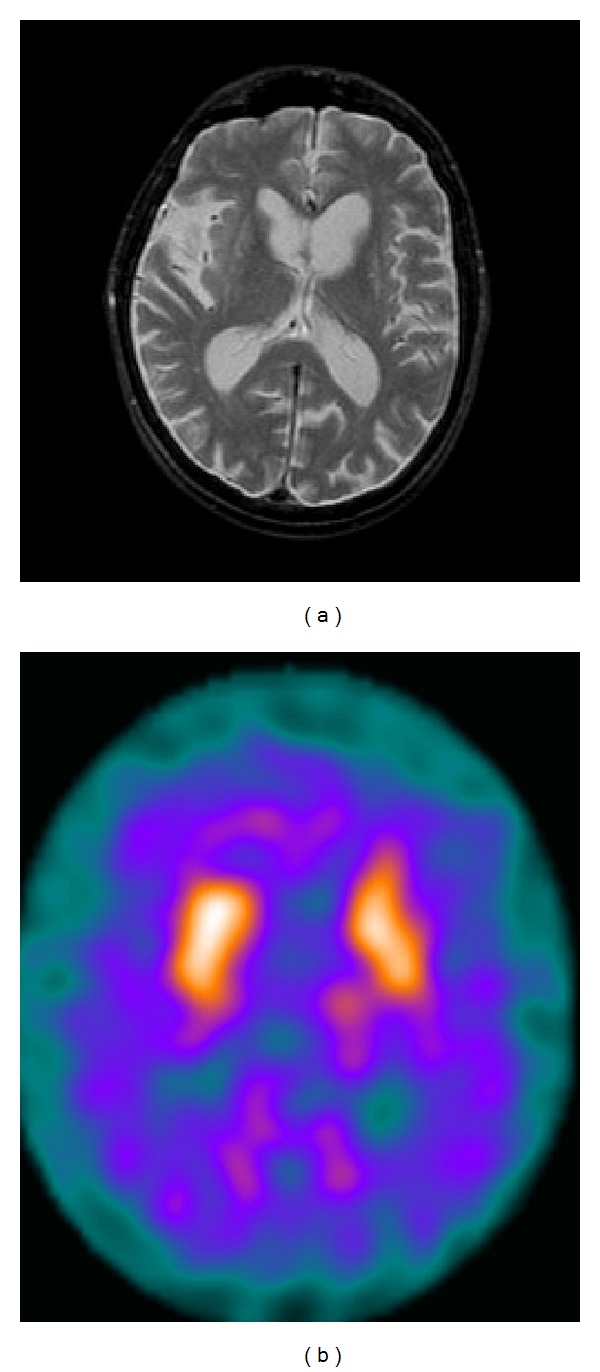
(a) Brain MRI showing severe atrophy of both caudate nuclei and moderate cortical atrophy. (b) SPECT imaging of the striatal dopamine transporter (DAT) with [^123^I]-*β*-CIT revealing a symmetric impairment of uptake in the caudate (mean 0.78, normal range 1.7–3.3) and putamen (mean 0.61, normal range 1.4–3.1).

Brain MRI showed severe atrophy of both caudate nuclei and moderate cortical atrophy. SPECT imaging of the striatal dopamine transporter (DAT) with [^123^I]-*β*-CIT revealed a symmetric impairment of uptake in both the caudate (mean 0.78, normal range 1.7–3.3) and putamen (mean 0.61, normal range 1.4–3.1) (Figures 1(a) and 1(b)). 

The history of choreic movements in the patient's brother and the MRI findings raised a clinical suspicion of HD. The patient gave his informed consent to be tested for HD, and molecular analysis disclosed 40 CAG repeats.

To improve the patient's motor function, pramipexole was started at 1.05 mg/day. The treatment resulted in a moderate improvement of bradykinesia and a decrease in festination and propulsion without worsening chorea and dystonia. Four months later, his UPDRS motor score and UHDRS motor scale were, respectively, 15 and 38. BDI showed a significant improvement in depression (BDI score = 3). 

Nonetheless, one year later, his walking progressively began to worsen. A further increase in pramipexole dosage was tried (2.10 mg/day), but severe neuropsychiatric side effects appeared (hallucinations and agitation). Therefore, pramipexole was stopped and replaced by L-Dopa, titrated up to 600 mg/day over three months. The neuropsychiatric side effects disappeared, but the patient's motor function improved a little. 

Over the following two years, his gait progressively worsened until in July 2008 he was no longer able to walk.

## 3. Discussion

Rigidity and bradykinesia are rare manifestations at HD motor onset although they commonly develop during the course of typical HD cases in which chorea is the presenting symptom. Moreover HD patients who present an akinetic and rigid form in the early disease stage have larger CAG expansions and an earlier age at onset than HD patients with typical choreic onset [3–5].

Our report describes a patient with a 40 CAG repeat in the HD gene and onset after age 50 whose presenting symptom was a progressive symmetrical Parkinsonism with severe gait difficulty associated with very few choreic movements. To our knowledge, only one report in the literature described late onset HD patients presenting with L-Dopa-responsive Parkinsonism [6]. In our opinion, these rare late onset HD presentations are worth describing, as similar cases could be misdiagnosed as vascular, toxic, or drug-induced parkinsonism or atypical neurodegenerative Parkinsonism (e.g., progressive supranuclear palsy or multiple system atrophy). Moreover, given that these late-onset patients typically have short CAG expansions even in the reduced penetrance range, the prompt diagnosis of these cases may have implications for genetic counseling in their families. 

The development of Parkinsonism in HD patients causes a progressive functional disability [7]; thus, a better knowledge of the pathophysiological mechanisms underlying parkinsonism (i.e., pre- or postsynaptic nigrostriatal degeneration) in HD may provide therapeutic strategies. Early studies suggested that bradykinesia in HD results from a massive degeneration of the striatal neurons (expressing the D1 receptor) projecting to the globus pallidus interna (the so-called “direct pathway”), associated with relative preservation of the nigrostriatal pathway [8]. However a PET study highlighted a significant reduction of the presynaptic dopamine transporter density in five HD choreic patients [9]. Another PET study based on vesicular monoamine transporter type-2 (VMAT2) binding confirmed that nigrostriatal pathology occurs in HD, being particularly severe in the rigid form of the disease [10]. More recently, these data were further confirmed by an SPECT study (using ^123^I-Ioflupane to evaluate the presymptomatic dopaminergic system) that showed DAT binding changes in the striatum mainly at putamen level [11]. The ^123^I-Ioflupane SPECT study in our patient is consistent with these findings, showing a remarkable reduction in DAT binding in caudate and putamen. This nigrostriatal degeneration could account for his Parkinsonism and also explain the partial benefit he received from dopaminergic stimulation. However, the progressive loss of efficacy of the dopaminergic stimulation may depend on the progressive degeneration of the striatal neurons typical of HD.

In conclusion, this case illustrates that atypical parkinsonism can be the presentation symptom in HD late onset patients. Thus, genetic testing for HD should be considered in the presence of atypical Parkinsonism in elderly patients whose family history includes choreic disorders. Furthermore, the dopaminergic stimulation should be tried in these rare cases, giving particular attention to possible psychiatric side effects.
